# The Sound Generated by Mid-Ocean Ridge Black Smoker Hydrothermal Vents

**DOI:** 10.1371/journal.pone.0000133

**Published:** 2006-12-27

**Authors:** Timothy J. Crone, William S.D. Wilcock, Andrew H. Barclay, Jeffrey D. Parsons

**Affiliations:** School of Oceanography, University of Washington, Seattle, Washington, United States of America; Atlantic Oceanographic and Meteorological Laboratory, United States of America

## Abstract

Hydrothermal flow through seafloor black smoker vents is typically turbulent and vigorous, with speeds often exceeding 1 m/s. Although theory predicts that these flows will generate sound, the prevailing view has been that black smokers are essentially silent. Here we present the first unambiguous field recordings showing that these vents radiate significant acoustic energy. The sounds contain a broadband component and narrowband tones which are indicative of resonance. The amplitude of the broadband component shows tidal modulation which is indicative of discharge rate variations related to the mechanics of tidal loading. Vent sounds will provide researchers with new ways to study flow through sulfide structures, and may provide some local organisms with behavioral or navigational cues.

## Introduction

Mid-Ocean ridge hydrothermal systems support rich communities of chemosynthetic organisms and are conduits for large heat and chemical exchanges between young oceanic lithosphere and the ocean. On a global scale the time-averaged hydrothermal heat flux and many chemical fluxes are well constrained [1]. On local scales these fluxes are temporally and spatially variable [2]–[4], but the variations are poorly quantified because there are few time-series measurements of fluid flow with which to integrate temperature and chemical observations. While time-series measurements of flow have been obtained in low-temperature vents [4], [5], and point measurements have been obtained in black smokers [6], [7], no time-series measurements of black smoker flow exist. High temperatures, low pH, and mineral precipitation limit the long-term effectiveness of invasive flow measurement techniques commonly employed in these environments.

The development of a non-invasive flow measurement technique could solve this problem and enable the collection of extended time-series flow data. One proposed method [8] would use passive acoustic measurements and capitalize on the potential for fluid flow to produce sound [9]. Passive acoustic measurements near black smokers could provide flow rate information if flow-related sounds can be detected, and if a relationship between flow rate and acoustics can be established. Point measurements of flow using an invasive measurement technique [6] could be used to convert time-series measurements of acoustically-determined relative flow rates into absolute measurements.

While previous studies have noted an apparent increase in ambient noise within several hundred meters of two hydrothermal vent sites [10], [11], another study found no conclusive evidence that hydrothermal vents generate sound [8]. In this report we present the first detailed description of the localized sound generation by two mid-ocean ridge black smoker hydrothermal vents. We discuss the likely sound source mechanisms that operate to produce both broadband and narrowband signals. We then discuss the tidal variations observed in one record, which we argue is related to tidal forces affecting fluid circulation within the hydrothermal system. We conclude with speculation on the biological implications of black smoker sound production.

## Results

**Figure 1:**
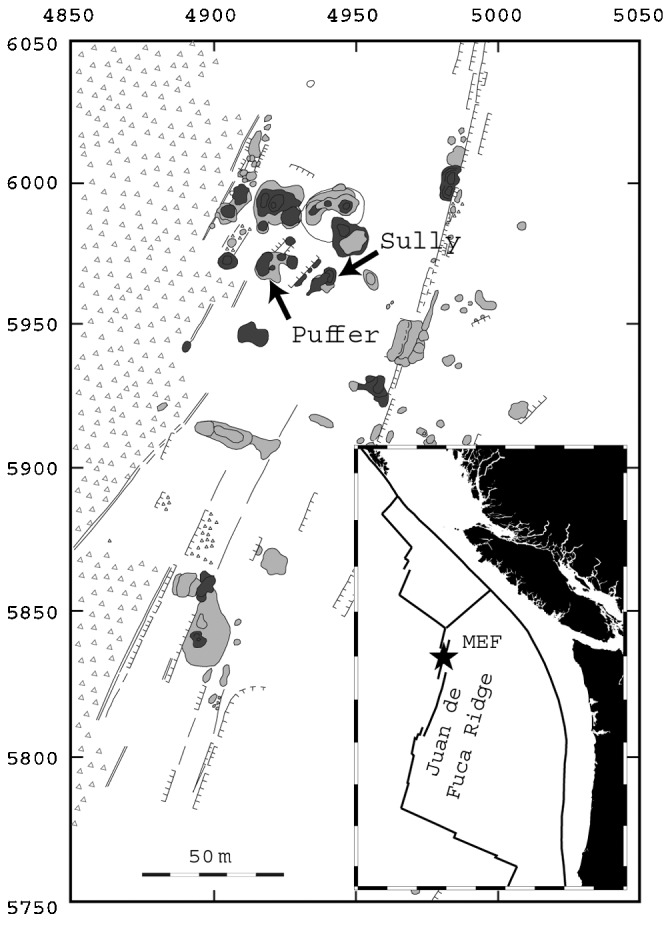
Geological map of the southern part of the Main Endeavour vent field, adapted from [43]. The acoustic recording system was deployed at the Sully and Puffer vents in September 2004 and 2005 respectively. On this map, dark gray objects represent active sulfide structures; light gray objects represent inactive sulfides or diffuse vents; triangles depict talus at the base of the west axial valley wall; and thin lines indicate faults and fissures. Inset shows the location of the Juan de Fuca Ridge in the northeast Pacific Ocean, approximately 500 km west of Seattle, WA, with the location of the Main Endeavour Field (MEF) marked with a star. Surrounding plate boundaries are also depicted.

**Figure 2:**
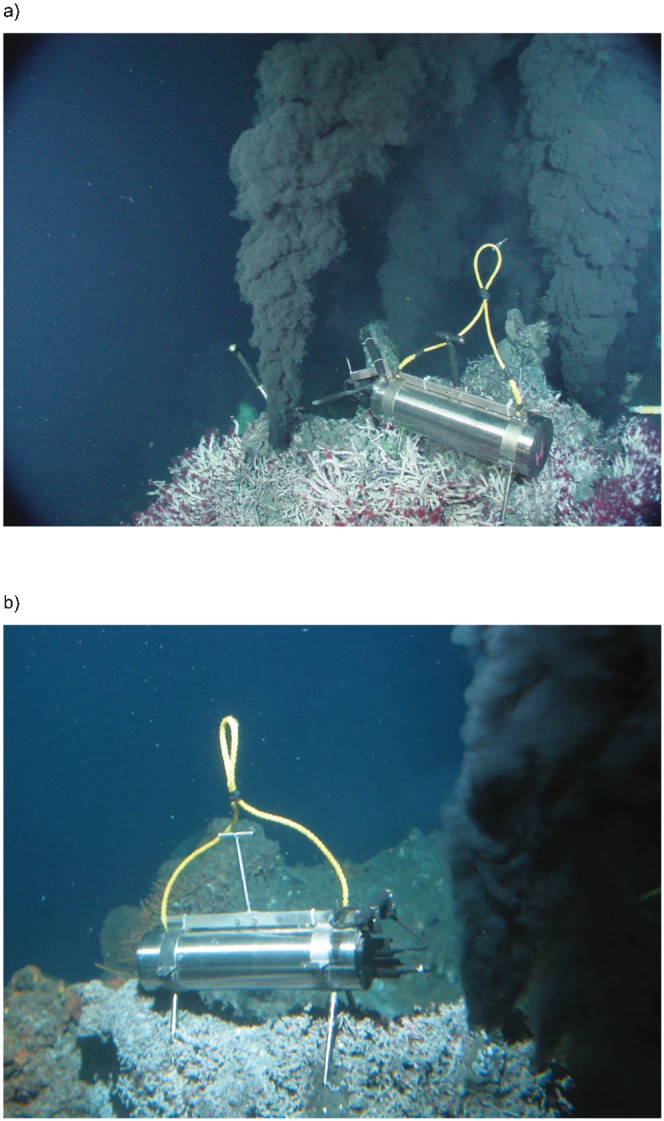
Photograph showing a) the first-generation digital acoustic recording system deployed at the Sully vent in September 2004, and b) the second-generation system deployed at the Puffer vent in September 2005. The cylindrical titanium pressure case housed the battery and recording electronics. The “case” hydrophone was attached to the stand assembly just above the bulkhead connectors at one end of the case. The bracket which held the “remote” hydrophone can be seen behind the black smoker jet in the center of a).

Using a stand-alone deep-sea digital acoustic recording system, we recorded 45 hours of continuous sound sampled at 1000 Hz in 2004, and 136 hours of continuous sound sampled at 1920 Hz in 2005, from the “Sully” and “Puffer” vents respectively (Figure 1). These two vents are situated approximately 2200 m below the sea surface, within the Main Endeavour vent field on the Juan de Fuca Ridge. We also recorded the local ambient noise field at a distance of ∼150 m from the nearest black smoker in 2004, and ∼25 m from the nearest black smoker in 2005. Movie S1 and Audio S1 contain samples of the recorded audio, and Figure 2 shows the recording system deployed at Sully and Puffer.

**Figure 3:**
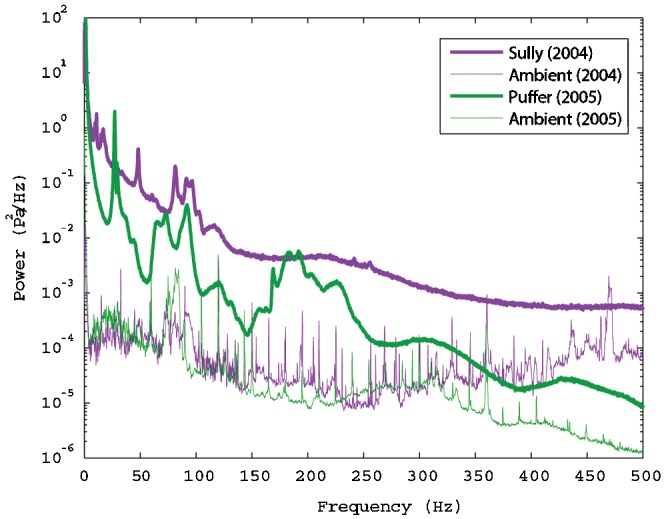
Typical hour-average power spectra derived from the two vent recordings, and the ambient noise recordings. Sharp peaks on the ambient spectra are associated with a nearby research vessel.

The power spectra of the recorded signals show that both vents radiate significant acoustic energy at all frequencies up to the anti-aliasing filter which has a corner frequency of 500 Hz (Figure 3). Both vents generate a broadband acoustic signal with power levels ∼10–30 dB above the ambient noise level. Both vents also produce numerous narrowband tones at frequencies ranging from ∼10–250 Hz, with power levels ∼10–20 dB above the broadband signal level and bandwidths of ∼5–15 Hz. Root-mean-square pressure fluctuations associated with these spectra, computed from the integral of these curves over the frequency range 5–500 Hz, are shown in Table 1.

**Table 1:**
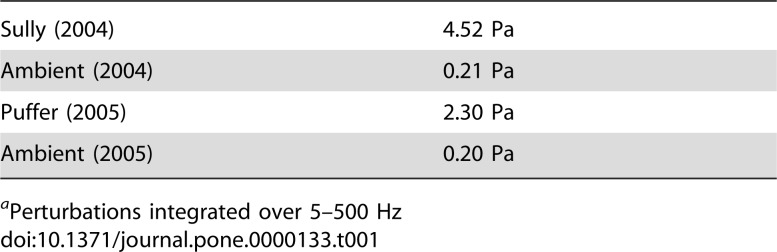
Root-Mean-Square Pressures^a^.

Sully (2004)	4.52 Pa
Ambient (2004)	0.21 Pa
Puffer (2005)	2.30 Pa
Ambient (2005)	0.20 Pa

a Perturbations integrated over 5–500 Hz

**Figure 4:**
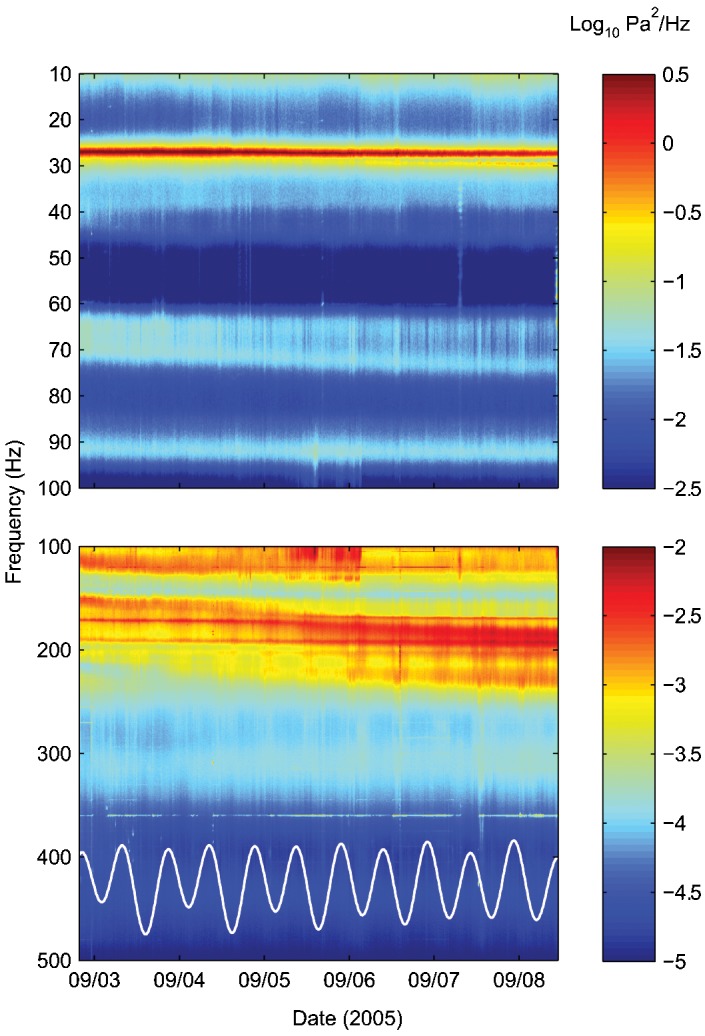
Spectrogram showing the temporal evolution of Puffer's power spectrum, based on 10-minute average spectra. Upper and lower panels have different color scales and vertical scales. Overlain on an arbitrary scale in the lower panel is the predicted tidal height for the study area [44]. The peak-to-peak tidal amplitude is ∼2 m. Tidal variability is discernible between ∼200–250 Hz for this choice of color scales. The very narrow peak at 360 Hz is associated with a nearby research vessel.

A spectrogram generated from the Puffer recording (Figure 4) and an animation of Puffer's acoustic power spectrum (Movie S2) illustrate the temporal evolution of this vent's acoustic signature. The general shape of the spectrum remains relatively constant, but some fine-scale features vary. For example, the spectral peaks centered at ∼27 Hz and ∼67 Hz each split into two separate peaks, and the power spectrum within the 150–250 Hz band changes significantly over the measurement period.

**Figure 5:**
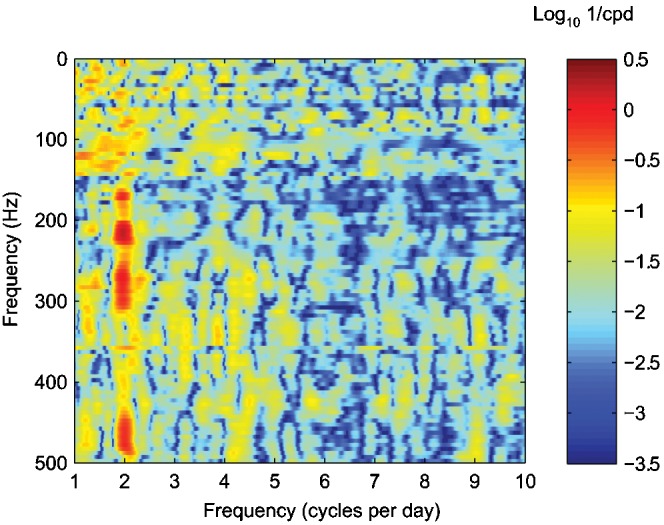
Contours of the spectra computed from the average spectral power of Puffer's acoustic signal in 5-Hz bands. The power level time-series have been normalized by their root-mean-square, thus the units of the contoured values are inverse cycles per day (cpd), and the relative magnitudes of the contours are only meaningful in the horizontal direction. The length of the time-series (136 hours) renders spectral information below ∼1 cycles per day unresolved. Semi-diurnal (∼1.95 cycles per day) variability of Puffer's acoustic power occurs within the ∼150–325 Hz and ∼450–500 Hz frequency bands.

**Figure 6:**
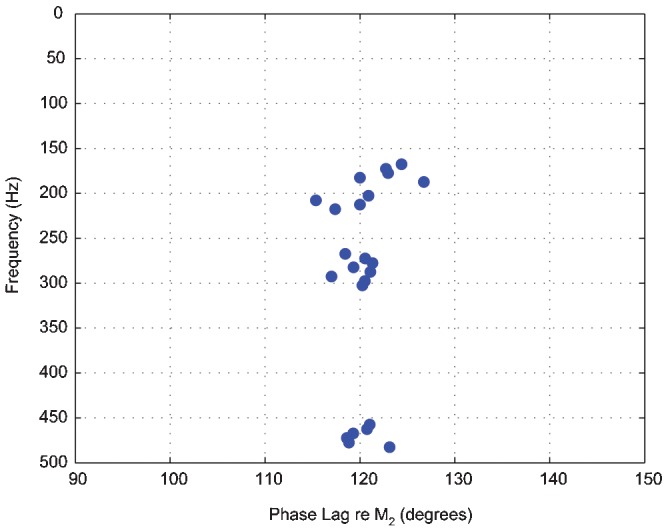
Phase lags of the maximal acoustic power output, relative to the M_2_ tidal component period of 12.42 h, for parts of the acoustic spectrum showing strong semi-diurnal periodicity (Figure 5). Phase lags cluster around 120°, or just over 4 h. Similar phase lags are predicted by numerical models of tidal loading on poroelastic hydrothermal convection systems [29].

Tidal variability is also evident in the acoustic record of Puffer. A comparison of the predicted tidal heights with the spectrogram (Figure 4) hints at this variability. For the choice of color scales in this figure, the power levels within the ∼200–250 Hz band appear elevated after high tide. A spectral analysis of the power in different frequency bands reveals that the acoustic signals contain semi-diurnal periodicity within the ∼150–325 Hz and ∼450–500 Hz bands (Figure 5). The ∼1.95 cycles per day signal corresponds closely to the 1.93 cycles per day frequency of the 12.42-h M_2_ tidal component [12]. Cross correlation of the tidal signal with the time-series of power level in these frequency bands shows that maximal acoustic output lags the M_2_ tidal component by ∼115–127° (Figure 6). This is equivalent to a lag of ∼238–262 minutes with respect to high tide.

## Discussion

### Source Mechanisms

There are a variety of sound source mechanisms that could potentially operate within black smoker systems. The presence of both broadband and narrowband components in the acoustic signals indicate that multiple mechanisms are operating within Sully and Puffer. Potential broadband sources include boiling, cavitation, turbulent shear, advected fluid heterogeneity, pulsating exit flow, fluid–structure interactions, and volume changes associated with the cooling of hydrothermal fluid. Many of these source mechanisms were discussed in the context of black smoker systems in [8]. Here we review some of this work in light of our results.

Sound source mechanisms are typically divided into three separate classes of acoustic radiators: monopoles, dipoles, and quadrupoles. Each of these source types has a different near-field radiation pattern, where the near-field is defined as the region within one acoustic wavelength of the sound source [8]. Monopoles radiate sound through volume or mass fluctuations, and have one-dimensional pressure fields. This type of acoustic radiation would be generated by a sphere vibrating in its first mode, expansion and contraction [13]. In the near-field, pressure perturbations generated by a monopole fall off in proportion to 1/*r*, where *r* is the distance from the source. Dipole radiation usually arises from force fluctuations at a fluid–fluid or fluid–solid interface, and has a two-dimensional pressure field. This type of acoustic radiation would be generated by a rigid sphere oscillating side to side, or by two monopoles spaced closely together and vibrating out of phase [13]. In the near-field, pressure perturbations generated by a dipole fall off in proportion to 1/*r* ^2^. Quadrupole radiation arises from the fluctuating shear stresses within a turbulent fluid [14] and has a three-dimensional pressure field. This type of acoustic radiation would be generated by a sphere vibrating in its third mode, ellipsoidal distortion, or by two dipoles of opposite polarity situated side-by-side [13]. In the near-field, pressure perturbations generated by a quadrupole fall off in proportion to 1/*r* ^3^.

Boiling produces monopole acoustic radiation as the expansion, and sometimes the subsequent collapse, of vapor bubbles within a heated fluid produce pressure fluctuations that propagate away from the forming bubble [15]. While this mechanism could be a significant source of sound in vents that are boiling [8], the temperatures of Sully and Puffer were about 15–20°C below the ∼375°C boiling point of hydrothermal fluid at 2200 m depth [16] when we recorded their sound. Thus it is unlikely that boiling is a source of sound in these recordings.

Similar to boiling, cavitation also produces monopole radiation during the formation and collapse of vapor pockets. These pockets form in response to hydrodynamic pressure drops that bring local fluid pressures below the vapor pressure [17]. Cavitation can be a significant source of sound when it occurs [17], however the fluids issuing from Sully and Puffer are far from the two-phase curve [16], and are unlikely to cavitate as a result of hydrodynamic forces within these two chimneys [8].

Free turbulence in a fluid generates quadrupole acoustic radiation which is associated with the fluctuating shear stresses in the flow [14]. The power output from this mechanism is typically quite small in low Mach number flows [18], [19]. Near-field pressure perturbations associated with this mechanism can be approximated by [8]:
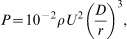
1where *P* is the root-mean-square pressure fluctuation, *r* is the fluid density, *U* is the mean fluid velocity, *D* is the vent orifice diameter, and *r* is the distance from the source. Letting *r* = 625 kg/m^3^ [16], *U* = 1 m/s, *D* = 0.05 m, and *r* = 0.5 m, we find that pressure fluctuations associated with free turbulence may equal ∼6×10^−3^ Pa. Such pressures are far below the ambient sound pressures recorded at the Main Endeavour field (Table 1), thus this source mechanism is not likely responsible for any of the acoustic radiation recorded in this study.

Turbulent fluid flows containing heterogeneous density or compressibility fields can generate significantly more sound than uniform fluid flows [20]. Pockets of differing density or compressibility can interact with hydrodynamic pressure variations to produce dipole acoustic radiation with wavelengths that are much longer than the heterogeneity length scale [20]. In black smoker systems, this can result in pressure fluctuations that are a factor of ∼4×10^2^ greater than those generated by turbulent shear in a uniform flow [8]. Thus pressure perturbations generated by this source mechanism may equal ∼2.4 Pa, which is similar in magnitude to the pressures measured near Sully and Puffer (Table 1), and suggests that fluid heterogeneity may play a role in sound production in these vents.

Mass flux variations at the vent orifice caused by pulsating flow can produce monopole acoustic radiation [18], [19]. This source mechanism functions much like a baffled piston [17], and the sound from this mechanism would originate from the plane of the vent orifice [18], [19]. Pressure perturbations associated with this mechanism can be approximated by [8]:
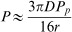
2where *P_p_* is the root-mean-square pressure fluctuation of the pulsating flow. Letting *P_p_* equal 10 percent of the total mean pressure, which can be approximated by *ρU* ^2^ [8], and letting *r* = 625 kg/m^3^ [16], *U* = 1 m/s, and *r* = 0.5 m, we find that pressures from pulsating flow may equal ∼3.7 Pa. Pressures of this magnitude would be easily detected over the background noise, and are indeed similar to those measured at both vents (Table 1). Thus pulsating flow may contribute to the sound signals recorded in this study.

The interaction of unsteady flow with the internal walls of the chimney can create force fluctuations at the fluid–solid interface, which can, in turn, cause the structure to vibrate and emit dipole acoustic radiation [9]. Sound from this mechanism would originate from within the chimney structure. It is difficult to predict the magnitude of acoustic pressure perturbations associated with source, as it will depend on many factors including the stiffness of the chimney and the geometry of the fluid conduits. However, this mechanism is strongly dependent on fluid flow, with acoustic intensities being proportional to roughly the fifth power of the mean flow rate [17], and coupling between the fluid and solid increasing with increased conduit roughness and tortuosity. Considering the rapid flow rates and the rough and often tortuous fluid pathways found in black smoker structures [21], we consider it possible that this sound source is significant in these systems.

Another potential sound source mechanism is related to fluid volume changes driven by the mixing of hydrothermal fluid with seawater. The equation of state for hydrothermal fluid at high temperatures and pressures predicts a significant volume decrease when this fluid mixes and exchanges heat with ambient seawater [16]. This process will produce monopole acoustic radiation with the source located in the jet mixing region some distance above the vent orifice. To the best of our knowledge, this sound source mechanism has not been investigated, thus there is no theory to quantify the magnitude of the pressure perturbations it may generate. However, considering the large density differences between hot and cold hydrothermal fluid [16], we consider it possible that this source is significant in these systems. Small-scale laboratory experiments could be used to investigate this mechanism and determine its sound production potential.

Thus we have identified four potential source mechanisms that might generate the broadband acoustic signals measured in this study, each of which being of a different mechanism class, and or having a different locus. Pulsating flow would generate monopole acoustic radiation with its locus situated at the vent orifice. Volume changes associated with the cooling of hydrothermal fluid would also generate monopole radiation, but with its locus situated in the mixing region of the jet. Fluid heterogeneity would generate dipole acoustic radiation with its locus situated in the mixing region of the jet. And fluid–structure interactions would generate dipole acoustic radiation with its locus situated within the chimney structure. The deployment of a hydrophone array in the near-field may therefore help discriminate between these possible sources by measuring the fall-off rate of the pressure fluctuations and by locating the sound sources.

Potential narrowband sources within black smokers are related to the excitation of the acoustic modes of resonators by unsteady flow. Such resonance typically involves flow past cavities, or flow impinging upon solid bodies. Among the many possible resonators are Helmholtz resonators, half-wave or quarter-wave resonators, and solid structures such as tubes, plates, or cavities within the chimneys [22]–[24]. Considering the typical acoustic properties of black smoker fluids, and the geometry of these structures, the frequencies of the observed resonant signals are reasonable. For example, the fundamental frequency *f* of a Helmholtz resonator is given by [9]:
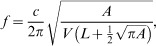
3where *c* is the speed of sound in the fluid filling the cavity, *A* is the area of the cavity opening, *V* is the volume of the cavity, and *L* is the length of the cavity opening. For a relatively small 2-liter cavity connected to the chimney conduit by an opening of diameter 0.02 m and a length of 0.04 m, filled with hot hydrothermal fluid for which *c* = 450 m/s [16], *f* would equal ∼120 Hz. For a quarter-wave oscillator, the first resonant mode is given by [23]:
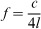
4where *l* is the length of the cavity. Thus for a 1-m tube closed at one end and filled with hot hydrothermal fluid, the fundamental frequency is ∼113 Hz.

Both Sully and Puffer produce acoustic tones at several different frequencies. The different tones might be produced by different types of resonators, or by several resonators of a single type but with different geometries and different relationships to the fluid flow. In either case, the tones that are generated will depend strongly on the vent's morphological structure, and each vent within the vent field is likely to have its own unique acoustic signature.

### Temporal Variability

The relatively long timescale changes observed in the broadband and narrowband signals could be related to a variety of factors. Changes in fluid temperature or chemical composition could affect the density or compressibility of the fluid which are both critical parameters for all of the above-mentioned acoustic source mechanisms, and could affect both the amplitude and frequency content of the acoustic signals. Such changes in fluid temperature and composition are common in these systems [25]–[28]. Changes in fluid flow rate can also affect acoustic amplitudes and frequencies for several of the above-mentioned source mechanisms, such as fluid–structure interactions [17]. Changes in the geometry of the vent structure could also affect the acoustic signals. Either through episodic cracking events, or through gradual mineral deposition/dissolution, channels and voids within the structure may change shape, thereby affecting relative flow rates through the chimney, and the fundamental resonant frequencies of any resonant bodies. Such changes in vent geometry have been observed on these timescales [21].

The tidal variations in the Puffer record are likely related to changes in fluid discharge rate. The strongest evidence for this is the observed lag in acoustic output relative to high tide (Figure 6). Recent two-dimensional numerical models of tidally forced poroelastic convection within mid-ocean ridge hydrothermal systems predict that maximal discharge rates will lag high tide by ∼125° [29]. Similar one-dimensional analytical models predict a lag of 135° [30]. Thus our observations are close to the expected values if it is assumed that faster discharge results in higher acoustic output—a good assumption for many of the potential acoustic source mechanisms. Other evidence shows that the tidal signal is not related to ocean currents, which are another conceivable source of tidal variability. We do not observe a ∼4 cycles per day signal which would be associated with local current speeds that peak ∼4 times per day [31]. We also do not observe a ∼1.5 cycles per day signal which would be associated with the motions of regional inertial currents [32]. Thus it is likely that changes in discharge rate are reflected in the acoustic record, and further investigation into passive acoustic flow measurement techniques is warranted.

It is not entirely clear why the observed tidal variations are confined to the ∼150–325 Hz and ∼450–500 Hz frequency bands. We suspect that the acoustic power in these bands is dominated by source mechanisms that are more sensitive to flow rate, such as fluid–structure interactions, which can have intensities that depend on the fifth power of the fluid velocity [17]. The sound in the other parts of the spectrum could be generated by a source mechanism that is not as strongly dependent on mean discharge rate, such as pulsating exit flow [17]. This hypothesis could be tested with an array of hydrophones capable of determining which sound source mechanisms dominate in different frequency bands, by locating the sound sources in different bands, and measuring the near-field pressure distribution in different bands.

### Biological Implications

Sound production by black smokers has possible implications for local organisms. Considering the near-field sound pressure fall-off predicted for the likely sound source mechanisms, we estimate that vent sound levels would be above ambient levels at a distance of ∼5–15 m from the vent orifice at the time our measurements were made. Thus fish, crustaceans, and cephalopods, which are common in these environments [33], [34] and can typically detect and process sound [35]–[38], might utilize this source of environmental information to their advantage. The acoustic detection of vent locations could help an organism avoid damage from hot hydrothermal fluid, and could provide foraging or reproductive benefits by helping an organism find food or a mating partner. An analogous adaptation is suspected in the Mid-Atlantic Ridge shrimp species *Rimicaris exoculata* which may use infrared light to locate hydrothermal vents [39]. In reef settings, certain fish larvae are known to use environmental sounds in their search for settlement habitat [40]. Novel field or laboratory studies could be used to investigate the effects of vent sounds on the local animal community.

### Conclusion

Our study shows that high-temperature seafloor vents produce high levels of acoustic radiation which can provide valuable information about geological and physical processes occurring within these systems, and may provide animals with information about the environment they inhabit. Several new lines of inquiry regarding the acoustical, geophysical and biological implications of hydrothermal vent sounds should soon be explored.

## Materials and Methods

### Acoustic Recording System

We developed two versions of the deep-sea digital acoustic recording system used in this study. Both versions were based on the Persistor CF2 microcontroller and both had 4 GB of flash memory capable of storing ∼6–10 days of continuous sound data. Both versions could be equipped with two hydrophones, based on the Benthos AQ-2000 piezoceramic sensor element. One hydrophone was affixed to the instrument's titanium pressure case, and the other had a 3 m cable and was attached to a bracket allowing it to be positioned just a few centimeters from a black smoker vent orifice. We refer to these two hydrophones as the “case” and “remote” hydrophones, respectively. The first-generation system, used in 2004, sampled on three channels continuously at 1000 Hz with a 12-bit A/D converter and a fixed gain for each channel. The second-generation system, used in 2005, sampled on two channels continuously at 1920 Hz with a 16-bit A/D converter and a programmable gain. Additional information on the instrument specifications is shown in Table 2.

**Table 2:**
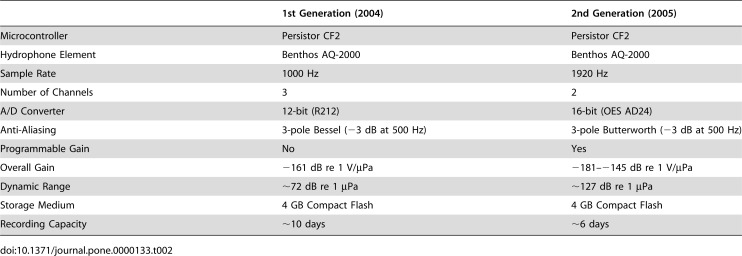
Recording System Specifications.

	1st Generation (2004)	2nd Generation (2005)
Microcontroller	Persistor CF2	Persistor CF2
Hydrophone Element	Benthos AQ-2000	Benthos AQ-2000
Sample Rate	1000 Hz	1920 Hz
Number of Channels	3	2
A/D Converter	12-bit (R212)	16-bit (OES AD24)
Anti-Aliasing	3-pole Bessel (−3 dB at 500 Hz)	3-pole Butterworth (−3 dB at 500 Hz)
Programmable Gain	No	Yes
Overall Gain	−161 dB re 1 V/µPa	−181–−145 dB re 1 V/µPa
Dynamic Range	∼72 dB re 1 µPa	∼127 dB re 1 µPa
Storage Medium	4 GB Compact Flash	4 GB Compact Flash
Recording Capacity	∼10 days	∼6 days

### Field Program

In September 2004 we lowered the acoustic recording system to the seafloor using a free-falling platform (called an elevator) which landed about 150 m east of the Sully vent. The instrument recorded several hours of ambient noise at this location. We then used a remotely operated vehicle to move the instrument to the Sully vent, with the case hydrophone positioned approximately 0.3 m from the vent orifice, and the remote hydrophone positioned approximately 0.1 m from the orifice. Figure 2a shows the instrument deployed at Sully. The acoustic output of this vent was higher than expected, and the remote hydrophone signal was heavily clipped on both channels. The case hydrophone was sufficiently sensitive to record the vent sounds, thus all the 2004 data shown in this paper were collected with the case hydrophone. After about 45 hours of data collection, the remote hydrophone cable was destroyed by hot hydrothermal fluid, at which time the instrument stopped recording.

In September 2005 we deployed the system at the Puffer vent with the case hydrophone positioned approximately 0.3 m from the vent orifice. The remote hydrophone had been destroyed by venting fluid in a previous deployment attempt during the same year, thus all of the 2005 data shown in this paper were collected with the case hydrophone. Figure 2b shows the instrument deployed at Puffer. After about 136 hours of data collection, the instrument was recovered, then placed on an elevator and returned to the seafloor to collect an ambient noise recording. The elevator landed about 25 m east of the Hulk vent in the northern part of the vent field, and at this location the instrument recorded several hours of data before being recovered.

### Data Reduction

We converted the raw hydrophone values into units of zero-mean pressure by first applying a 1-Hz high-pass 4-pole Butterworth filter. We divided the signal by the overall system gain used by the recording instrument for that channel, then applied the nominal sensitivity of the hydrophone element as published by the manufacturer.

We obtained 10-minute average spectra using Welch's method [41] with 2^15^-point fast Fourier transforms applied to 9600-point sections of the record. Each section of the record overlapped adjacent sections by 50 percent, and was multiplied by a Hamming window [42]. These 10-minute spectra were then time averaged to obtain the representative hour-average spectra shown in Figure 3. The 10-minute spectra are contoured in Figure 4.

We conducted the spectral analysis of the power time-series for different acoustic frequencies by first averaging the power into 5-Hz bands. For each of these records, we applied a 1-cycles-per-day high-pass 4-pole Butterworth filter, then normalized the record by its root-mean-square. Finally we multiplied each record by a 50-percent Tukey (cosine-taper) window [42], and computed periodograms using a 2^14^-point fast Fourier transform. Contours of these periodograms are shown in Figure 5.

## Supporting Information

Audio S1 Audio file containing a short section of sound collected with the black smoker acoustic recording system at Puffer in September 2005. The audio has been upsampled to 8 kHz, and high-pass filtered at 10 Hz using a 4-pole Butterworth filter. It is played in real-time (i.e. without time stretching or pitch shifting). Because much of the acoustic energy falls below ∼100 Hz, speakers with good bass response are required to properly reproduce the sound. Most laptop speakers will not produce sound.
(0.96 MB WAV)

Movie S1 Movie showing the black smoker acoustic recording system deployed at the Sully vent in September 2004 with audio from the same deployment. The audio and video are not contemporaneous because the remotely-operated vehicle carrying the video camera generated excessive noise. The video is included to provide context for the audio. The audio has been upsampled to 8 kHz, and high-pass filtered at 10 Hz using a 4-pole Butterworth filter. It is played in real-time (i.e. without time stretching or pitch shifting). Because much of the acoustic energy falls below ∼100 Hz, speakers with good bass response are required to properly reproduce the sound. Most laptop speakers will not produce sound.
(9.58 MB MOV)

Movie S2 Animation showing the temporal evolution of Puffer's acoustic power spectrum over the ∼5.6-day measurement period. The starting spectrum remains in the background for reference. Spectral peaks at ∼27 Hz and ∼67 Hz each split into two, and the spectrum between ∼150-250 Hz changes significantly. Tidal variability is discernible near 200 Hz. The very sharp spectral peaks that appear intermittently are associated with a research vessel which is sometimes within range of the acoustic recording system.
(3.43 MB MOV)

## References

[1] Elderfield H, Schultz A (1996). Mid-ocean ridge hydrothermal fluxes and the chemical composition of the ocean.. Annu Rev Earth Planet Sci.

[2] Baker ET (1994). A 6-year time series of hydrothermal plumes over the Cleft segment of the Juan de Fuca Ridge.. J Geophys Res.

[3] Delaney JR, Kelley DS, Lilley MD, Butterfield DA, Baross JA (1998). The quantum event of oceanic crustal accretion: Impacts of diking at mid-ocean ridges.. Science.

[4] Schultz A, Dickson P, Elderfield H (1996). Temporal variations in diffuse hydrothermal flow at TAG.. Geophys Res Lett.

[5] Pruis MJ, Johnson HP (2004). Tapping into the sub-seafloor: examining diffuse flow and temperature from an active seamount on the Juan de Fuca Ridge.. Earth Planet Sci Lett.

[6] Converse DR, Holland HD, Edmond JM (1984). Flow rates in the axial hot springs of the East Pacific Rise (21°N): Implications for the heat budget and the formation of massive sulfide deposits.. Earth Planet Sci Lett.

[7] Little SA, Stolzenbach KD, Von Herzen RP (1987). Measurements of plume flow from a hydrothermal vent field.. J Geophys Res.

[8] Little SH, Stolzenbach KD, Purdy GM (1990). The sound field near hydrothermal vents on Axial Seamount, Juan de Fuca Ridge.. J Geophys Res.

[9] Blake WK (1986). Mechanics of Flow-Induced Sound and Vibration,.

[10] Bibee LD, Jacobson RS (1986). Acoustic noise measurements on Axial Seamount, Juan de Fuca Ridge.. Geophys Res Lett.

[11] Riedesel M, Orcutt JA, Macdonald KC, McClain JS (1982). Microearthquakes in the Black Smoker hydrothermal field, East Pacific Rise at 21°N.. J Geophys Res.

[12] Melchior P (1983). The Tides of the Planet Earth..

[13] Powell A (1990). Some aspects of aeroacoustics: From Rayleigh until today.. J Vib Acoust.

[14] Lighthill MJ (1952). On sound generated aerodynamically: I. General theory.. Proceedings of the Royal Society of London Series A.

[15] Nesis Ye I (1991). Growth of vapor bubbles and the acoustic noise of a boiling liquid.. Fluid Mech Sov Res.

[16] Bischoff JL, Rosenbauer RJ (1985). An empirical equation of state for hydrothermal seawater (3.2 percent NaCl).. Am J Sci.

[17] Ross D (1976). Mechanics of Underwater Noise..

[18] Plett EG, Summerfield M (1974). Jet engine exhaust noise due to rough combustion and nonsteady aerodynamic sources.. J Acoust Soc Am.

[19] Plett EG, Summerfield M (1975). Erratum: “Jet engine exhaust noise due to rough combustion and nonsteady aerodynamic sources” [J. Acoust. Soc. Am. 56, 516–522 (1974)].. J Acoust Soc Am.

[20] Morfey CL (1973). Amplification of aerodynamic noise by convected flow inhomogeneities.. J Sound Vib.

[21] Koski RA, Jonasson IR, Kadko DC, Smith VK, Wong FL (1994). Compositions, growth mechanisms, and temporal relations of hydrothermal sulfide-sulfate-silica chimneys at the northern Cleft segment, Juan de Fuca Ridge.. J Geophys Res.

[22] Howe MS (1998). Acoustics of Fluid–Structure Interactions..

[23] Rockwell D, Naudascher E (1978). Review—self-sustaining oscillations of flow past cavities.. J Fluid Eng.

[24] Rockwell D, Naudascher E (1979). Self-sustained oscillations of impinging free shear layers.. Annu Rev Fluid Mech.

[25] Butterfield DA, Jonasson IR, Massoth GJ, Feely RA, Roe KK (1997). Seafloor eruptions and evolution of hydrothermal fluid chemistry.. Philosophical Transactions of the Royal Society of London A.

[26] Fornari DJ, Shank T, Von Damm KL, Gregg TKP, Lilley M (1998). Time-series temperature measurements at high-temperature hydrothermal vents, East Pacific Rise 9°49′–51′N.. Earth Planet Sci Lett.

[27] Johnson HP, Hutnak M, Dziak RP, Fox CG, Urcuyo I (2000). Earthquake-induced changes in a hydrothermal system on the Juan de Fuca mid-ocean ridge.. Nature.

[28] Lilley MD, Butterfield DA, Lupton JE, Olson EJ (2003). Magmatic events can produce rapid changes in hydrothermal vent chemistry.. Nature.

[29] Crone TJ, Wilcock WSD (2005). Modeling the effects of tidal loading on mid-ocean ridge hydrothermal systems.. Geochem Geophys Geosyst.

[30] Jupp TE, Schultz A (2004). A poroelastic model for the tidal modulation of seafloor hydrothermal systems.. J Geophys Res.

[31] Thomson RE, Mihály SF, Rabinovich AB, McDuff RE, Veirs SR (2003). Constrained circulation at Endeavour ridge facilitates colonization by vent larvae.. Nature.

[32] Thomson RE, Roth SE, Dymond J (1990). Near-inertial motions over a mid-ocean ridge: Effects of topography and hydrothermal plumes.. J Geophys Res.

[33] Desbruyères D, Segonzac M, Bright M (2006). Handbook of Deep-Sea Hydrothermal Vent Fauna,.

[34] Wolff T (2005). Composition and endemism of the deep-sea hydrothermal vent fauna.. Cah Biol Mar.

[35] Packard A, Karlsen HE, Sand O (1990). Low frequency hearing in cephalopods.. Journal of Comparative Physiology A: Neuroethology, Sensory, Neural, and Behavioral Physiology.

[36] Popper AN, Salmon M (2001). Acoustic detection and communication by decapod crustaceans.. Journal of Comparative Physiology A: Neuroethology, Sensory, Neural, and Behavioral Physiology.

[37] Schuijf A, Hawkins AD (1976). Sound Reception in Fish,.

[38] von Frisch K (1923). Ein Zwergwels, der kommt, wenn man ihm pfeift.. Biol Zentralbl.

[39] Van Dover CL, Szuts EZ, Chamberlain SC, Cann JR (1989). A novel eye in ‘eyeless’ shrimp from hydrothermal vents of the Mid-Atlantic Ridge.. Nature.

[40] Tolimieri N, Jeffs A, Montgomery JC (2000). Ambient sound as a cue for navigation by the pelagic larvae of reef fishes.. Mar Ecol Progr.

[41] Welch PD (1967). The use of fast Fourier transform for the estimation of power spectra: A method based on time-averaging over short, modified periodograms.. IEEE Trans Audio Electroacoust.

[42] Harris FJ (1978). On the use of windows for harmonic analysis with the discrete Fourier transform.. Proc IEEE.

[43] Delaney JR, Robigou V, McDuff RE (1992). Geology of a vigorous hydrothermal system on the Endeavour segment, Juan de Fuca Ridge.. J Geophys Res.

[44] Mofjeld HO, González FI, Eble MC (1995). Ocean tides in the continental margin off the Pacific Northwest Shelf.. J Geophys Res.

